# Stochastic tsunami modeling induced by kinematic complex sources

**DOI:** 10.1038/s41598-022-21336-8

**Published:** 2022-10-07

**Authors:** Mauricio Fuentes, Sebastián Riquelme

**Affiliations:** 1 Department of Geophysics, University of Chile, Santiago, Chile; 2 National Seismological Center, University of Chile, Santiago, Chile

**Keywords:** Geophysics, Natural hazards, Seismology

## Abstract

In this study, local tsunami hazard due to seismic sources is evaluated in a stochastic framework. Several assumptions such as static passive generation, constant rake angle and source centroid (among others) are relaxed. Spatial uncertainties are modeled in a large set of scenarios. The proposed methodology is easy to implement and can be combined with other types of sources or hazards. Application in the Kuril-Kamchatkah trench shows a straightforward use of our methodology, producing simple hazard maps, which can be replicated in any region of the world.

## Introduction

Tsunami hazard has been extensively addressed^1,2^. In general, tsunami hazards depend on the characterization of the probabilistic nature of the different tsunami sources, such as, earthquakes, landslides, volcanoes, impact of asteroids, among others. Grezio et al.^3^ reviewed PTHA for seismic and non-seismic sources including different methods for uncertainties quantification.

Depending on the specific objective, tsunami hazard assessment may consider just a single scenario in a deterministic approach, or a probabilistic framework in absence of complete information. Usually, in a deterministic case, an extreme case scenario is considered. Nonetheless, the feature “extreme” is established in a short time window or with little statistical history, which means that the considered event may not be “extreme” or having a neglected probability of occurrence. Thus, coastal planning, disaster risk management and reduction, and insurance companies rely on a strong characterization of the stochastic hazard.

**Figure 1:**
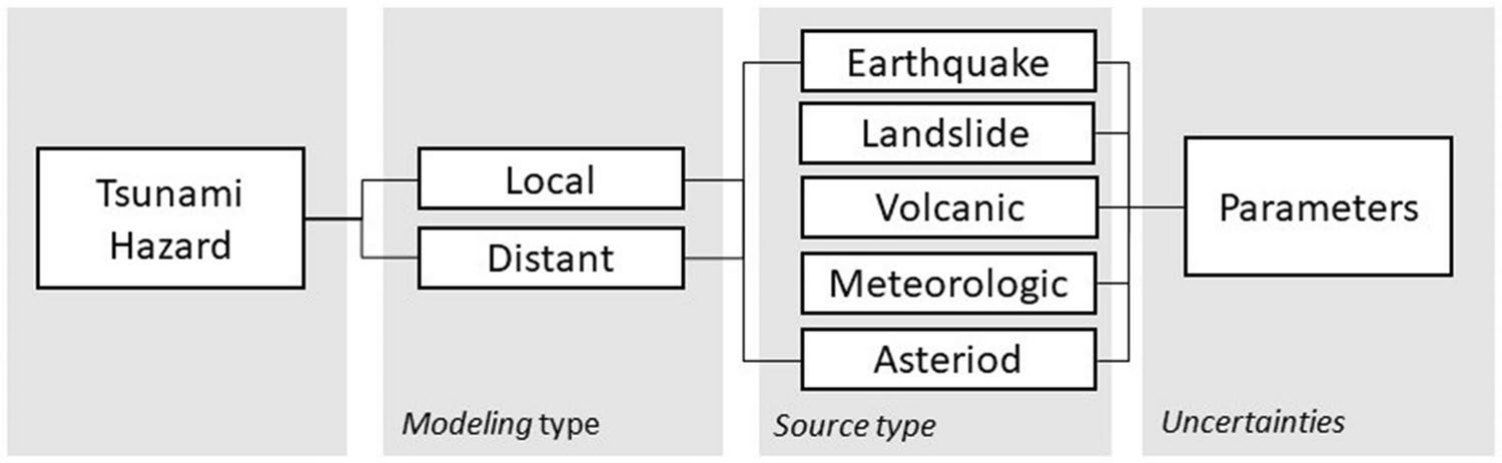
Diagram of tsunami hazard components.

Complete Probabilistic Tsunami Hazard Assessment (PTHA) considers all types of generating sources (Fig. 1). In relative terms, only earthquake source occurrence is better understood. For instance, Ravinobich^4^ describes the complexity to recognize and to catalog meteotsunamis, complicating the modeling of a probability of occurrence. Ward & Asphaug^5^ provides a simple approach to evaluate the tsunami hazard due to asteroid impacts. The major challenge is to overcome the lack of information about the annual rate of impacts of significant sizes. The authors proposed a power law in terms of radii of the objects. Løvholt et al.^6^ declared “*the probabilistic landslide tsunami hazard analysis (LPTHA) is still in its infancy*” and also stated the insufficient data cannot permit constrain annual rates, which mainly control LPTHA. Similarly, in the case of volcanoes, there are multiple ways in which a tsunami can be triggered^7^. According to the National Geophysical Data Center / World Data Service, from 4360 BC to today, 171 of 866 events of volcanic activity have produced a tsunami^8^. Nevertheless, many of them are associated with aerial or submarine landslides, being unclear where those events should be classified/counted. This introduces a large source of uncertainty. For example, if we assume that 90% of the tsunamis triggered by volcanic activity are landslide tsunamis, then the annual rate lies between 1/400 to 1/40.

It is well-known that the majority of tsunamis have been generated by large earthquakes in subduction zones. Around 70% (1942 of 2767) of all identified tsunamis are related to earthquake sources^1^.

Earthquake recurrence rates have been widely studied (e.g.,^9–14^). Roughly, earthquake recurrence periods follow power laws, where large earthquakes are exponentially less frequent than small ones. Parameters related with these laws are retrieved from extensive seismic catalogs.

To determine what zones are prone to release significant seismic moment, usually, the coupling index is employed^15,16^. By combining those tools, earthquake scenarios can be established^17–19^.

The last component of seismic scenarios is the generation of a realistic source. Seismic sources have plenty of ingredients that make their modeling challenging. In contrast to the traditional uniform slip models, real sources have patches, complexity, time dependence, dynamic parameters, among others. The way the source shows up, introduces spatial randomness.

The stochastic behavior of the seismic source has been addressed by several authors (^20–23^, among others). There are two equivalent ways in which static slip distributions are sampled. In the physical space, probability distribution of the slip is assumed with an imposed correlation matrix, which controls the patch sizes. This is useful for examining main modes through a Karhunen-Loève (KL) expansion^24^. By the other hand, in the wavenumber domain, the spatial slip distribution is expressed in complex polar form, where the power spectrum is modeled as a Von Karman filter, imposing a power law decay for high wavenumbers and a random phase. Both approaches are connected to each other via Fourier Transform properties.

To include as much as possible variability on the seismic source, kinematics rupture parameters should also be included. However, the influence of the kinematic part of the rupture often is neglected when tsunamis are modeled. Most of the time, passive tsunami generation is employed, which means that seafloor deformation is replicated into the sea surface. This is true under two assumptions: one is that the depth of the water is very small compared to the wavelength on the water surface and the rupture velocity is considered instantaneous compared to the tsunami velocity.

Kajiura^25^ first demonstrated that the tsunami can be considered instantaneous when the rupture velocity is faster than the tsunami velocity. Kanamori^26^ introduced the concept and the first observations of tsunami earthquakes. These are earthquakes that release energy in a low frequency band and are slower than regular earthquakes. The first tsunami earthquake detected by broadband seismometers was the Mw 7.7 1992 Nicaragua earthquake, this event had a rupture velocity of 0.8 to 1.1 km/s^27^, and the idea of very slow earthquakes started to emerge.

Yeo et al.^28^ evaluated the effect on tsunami runup due to the delayed multi-faults activation along the east coast of Korea, finding 2 to 42% of amplification in comparison with a static tsunami generation, declaring the importance of the source kinematics for certain cases. Studies regarding the source velocity were addressed, for instance, by Fuentes et al.^29^ and Riquelme & Fuentes^30.^

Here, we consider the source kinematics to include it in stochastic scenarios sampling with the aim to explore a wider range of rupture velocities. The proposed methodology is presented in “Methodology”, where descriptions of the used models and equations are provided. Also, a case study in the Kuril-Kamchatka trench is posed. In “Results”, results of the methodology are presented. Finally, “Conclusions” contains conclusions obtained from this study.

## Methodology

### Seismic source

First, for a given region, the slab 2.0 is used to grid the target area considering the uncertainties in depth^31^.

The spatial slip distribution $u\left( {x,y} \right)$ is introduced with power law decay in the wavenumber domain as proposed by Graves and Pitarka^23^,^32^:

1 $$\underline {u} \left( {k_{x} ,k_{y} } \right) = F\underline {D} \left( {k_{x} ,k_{y} } \right) + \left( {1 - F} \right)S$$

where:

$D\left( {x,y} \right)$ is a tapered uniform slip distribution with a given erage slip (associated with a given magnitude).

$$S\left( {k_{x} ,k_{y} } \right) = \frac{{\underline {D} \left( {0,0} \right)}}{{\sqrt {a_{x } a_{z } } }}K\left( {k_{x} ,k_{y} } \right)e^{{\varphi \left( {k_{x,} k_{y} } \right)}}$$.

$K\left( {k_{x} ,k_{y} } \right) = \frac{{\sqrt {a_{x } a_{z } } }}{{\left( {1 + (a_{x } k_{x } )^{2 } + (a_{z } k_{y } )^{2 } } \right)^{{0.5\left( {1 + H_{e} } \right)}} }}$ is the Von Karman spectrum with a Hurst exponent $H_{e}$.

$\varphi \left( {k_{x} ,k_{y} } \right)$ is a random phase, i.e., each angle is randomly selected in the interval $\left[ {0, 2\pi } \right],$ with a uniform distribution.

$F\left( {k_{x} ,k_{y} } \right) = \frac{1}{{1 + (Lk_{x } )^{2 } + (Wk_{y } )^{2 } }}$, $L,W$ are the fault dimensions.

Main parameters, such as average slip ${s}_{a}$, maximum allowed slip ${s}_{m}$, length $L ,$ width $W$, correlation lengths ${a}_{x }, {a}_{z}$ are taken from De Risi and Goda^33^, which account for uncertainties associated with the regression of scaling laws and also the correlation between them. The Hurst exponent is sampled from an approximated PDF given by Melgar et al.^34^ with a truncated normal distribution in the interval $\left[ {0.2; 0.8} \right] ,$ with a mean value of 0.36 and a c.v. of 50%. The slip distribution is then obtained by inverting $\underline{u}({k}_{x},{k}_{y})$ to the space domain.

Next, a point inside the source area is selected as a hypocenter. The selection is random, following the findings in Melgar and Hayes^34^. The along-dip coordinate (as a fraction of $W$) follows a normal distribution $N\left( {0.046; 0.208} \right)$, with origin in the middle of the fault (negative means up-dip). Similarly, the along-strike coordinate follows an exponential distribution, accounting for more probability on bilateral ruptures. Thus, a binomial(0.5) chooses the positive or negative strike half of the source, and en the fraction relative to the length from the middle of the source area follows an $Exp \left( {\frac{1}{5.435}} \right)$.

With a selected hypocenter, it is possible to introduce a rupture front to account for the main behavior of the kinematics of the source. To do this, the rupture time from the hypocenter to each point is calculated with a fixed average rupture velocity. This velocity is taken randomly from a uniform distribution in the interval $\left[ {v_{0} ; v_{1} } \right]$. Initially, this defines a perfect circular expanding rupture front. However, perturbations are included to make the rupture faster over the zones where the slip is higher^23^. Rise-time is also modeled as suggested by Graves and Pitarka^23^,^32^, which scales with square root of the (random) slip. The local slip-rate function is modeled with a Dreger slip-rate with a parameter of 0.2, as proposed by Melgar et al.^35^.

The last component to add in the kinematic stochastic source sampling, is to generate a rake angle distribution. This parameter is generally ignored or fixed for simplicity, usually, in tsunami modeling. Nonetheless, it is highly unlikely to have all the slip vectors pointing in the same direction. This assumption clearly contradicts the basic concepts of entropy. In order to include this parameter, an average rake value is fixed. Then, keeping the same spectrum correlation of the slip distribution, a random rake field is sampled. The resulting rake distribution keeps the coherency of the entropy, where statistically speaking, the slip vectors point to the same direction inside the slip patches and are uncorrelated otherwise.

The way to construct the rake field is similar to the slip distribution:

$$\alpha \left( {k_{x} .k_{y} } \right) = FD_{\alpha } \left( {k_{x} ,k_{y} } \right) + \left( {1 - F} \right)S_{\alpha }$$

where

$\alpha \left( {k_{x} .k_{y} } \right)$ is the final rake spectrum, $D_{\alpha }$ is a constant rake field, with a given value of $\alpha_{0}$ and $S_{\alpha } \left( {k_{x} ,k_{y} } \right) = \frac{{\alpha_{\sigma } }}{{\sqrt {a_{x } a_{z } } }}K\left( {k_{x} ,k_{y} } \right)e^{{\varphi \left( {k_{x,} k_{y} } \right)}}$.

Here, $\alpha_{\sigma }$ represents the rake standard deviation. It is important to recall that $\varphi \left( {k_{x,} k_{y} } \right)$ has to be the same random phase used for the slip distribution in order to keep the same correlation.

### Tsunami simulation

In order to include the source kinematics into the tsunami process, the numerical model NEOWAVE is employed^36,37^. This solver is a shock-capturing finite difference scheme that numerically solves the nonlinear shallow water equations in spherical coordinates, in a staggered grid. It also includes a non-hydrostatic component. To reduce computing time, NEOWAVE allows nesting different grids to focus the analysis on a specific coastal location. This nesting process must respect the CFL condition at all levels and permits to retrieve local parameters such as flow depth, inundation limits, run-up, moment flux, among others.

NEOWAVE requires two main inputs: the topo-bathymetric data and the seismic source parameters. The kinematic tsunami generation employs the Okada analytical solution^38^ and the horizontal contributions^39^. Each rectangular element is activated according to its rupture time, and it lasts its rise-time, depending on the temporal resolution of the numerical simulation.

**Figure 2:**
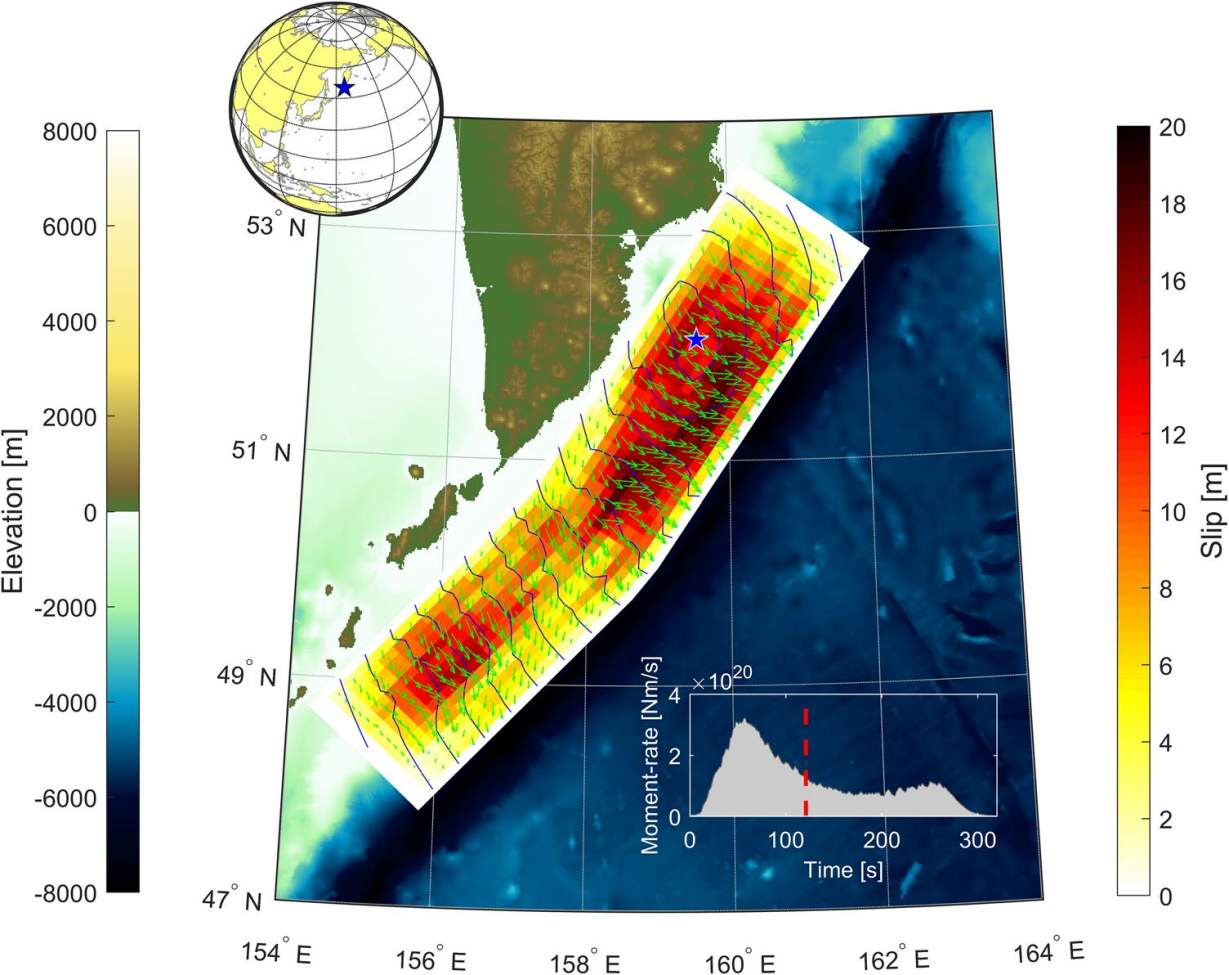
Realization of a complete kinematic field of a slip distribution. Green arrows depict rake directions and isochrones of the rupture front are shown each 20 s. The inset shows the source rate time function with its centroid time. (Figure created with MATLAB 2022a, https://www.mathworks.com/).

**Figure 3:**
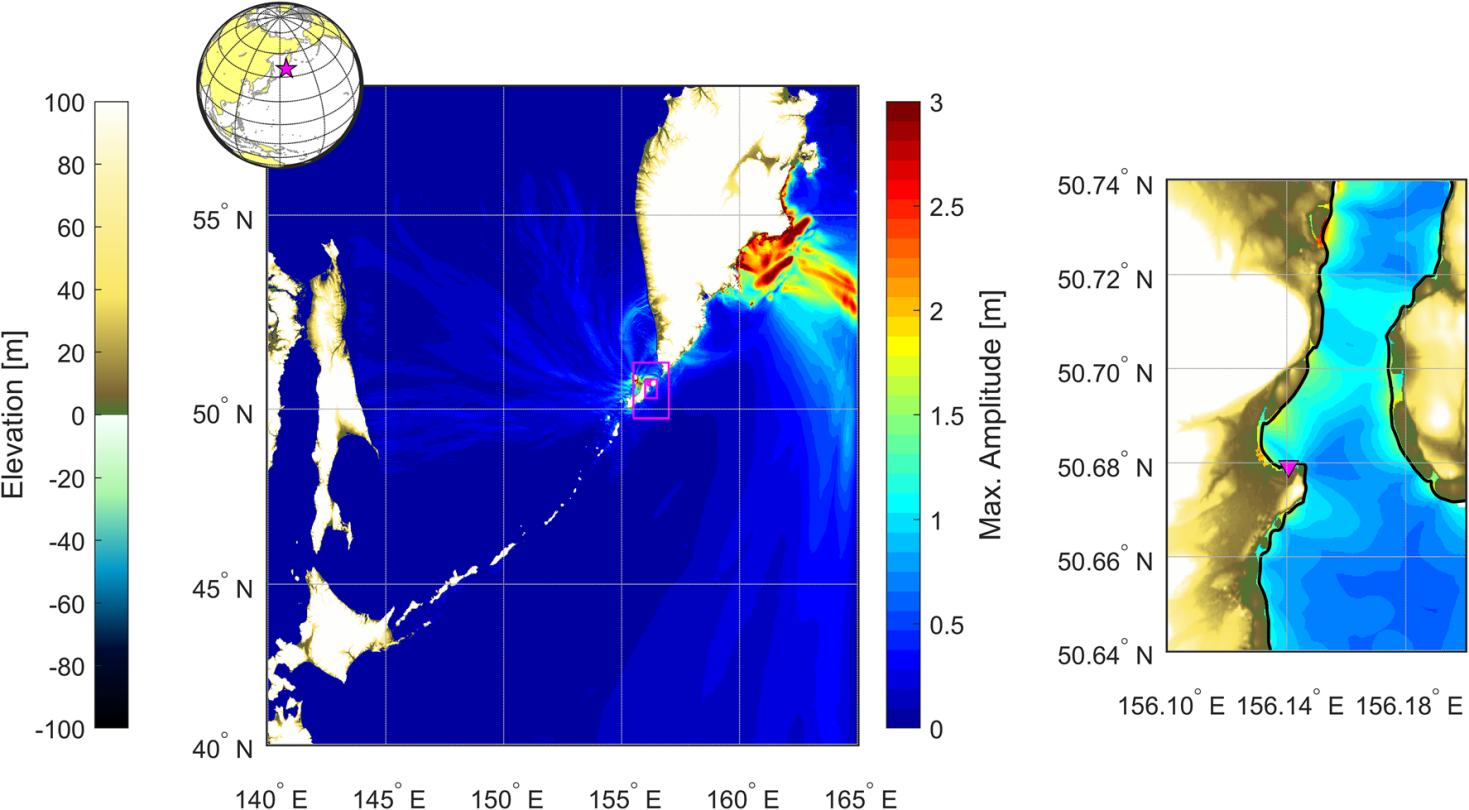
Example of a tsunami simulation with 4 nested levels. Magenta triangle marks the location of a virtual tide gauge.

Due to the nature of the gridded rupture area, some elements are not rectangles but trapezoids. To overcome this issue, each trapezoid is approximated with the best least square rectangle that minimizes the area difference^40^. This ensures to respect the given seismic moment without generating numerical artifacts. Example of a simulation with the sample shown in Fig. 2, is displayed in Fig. 3, where local inundation is computed with the numerical solver.

In a memoryless Poissonian process, the annual rate of exceedance of the intensity variable $I$ at location $x$ by $I_{0}$ is given by

2 $$\lambda (I\left( x \right) > I_{0} ) = \mathop \sum \limits_{e \in E }^{{}} P\left( {I\left( {x|e} \right) > I_{0} } \right)\lambda \left( e \right)$$

where $e$ is any event from a set of all possible events $E$. $\lambda \left( e \right)$ corresponds to the annual occurrence rate of the event $e$. $I\left( x \right)$ stands for runup height, moment flux, or flow depth, among others.

For a given exposure time $T$, the final probability of exceedance is given by an exponential distribution

3 $$P_{T} \left( {I\left( x \right) > I_{0} } \right) = 1 - exp( - \lambda (I\left( x \right) > I_{0} )T)$$

In the case of tsunamigenic earthquakes, $\lambda \left( e \right)$ can be associated with a magnitude and the G-R law^9^. Utsu^10^ proposed a modified G-R law with bounds, namely, Truncated G-R law, which is more suitable to avoid the subset of small earthquakes that fall out of the recording capabilities, and to restrict from infinite magnitudes.

4 $$\frac{d}{dm}N\left( m \right) : = n\left( m \right) = 10^{a - bm} , m_{1} \le m \le m_{2}$$

where $N\left( m \right)$ is the number of earthquakes of magnitude greater or equal to $m$ in a given time interval $\Delta T$, and $a, b$ are parameters determined with empirical data from seismic catalogs.

The occurrence rate, from the truncated Gutenberg-Richter law, is

5 $$\lambda \left( {e_{m} } \right) = \lambda_{{m_{1} }} \frac{{10^{ - bm} - 10^{{ - bm_{2} }} }}{{10^{{ - bm_{1} }} - 10^{{ - bm_{2} }} }}$$

where $\lambda_{{m_{1} }}$, $b$ and $m_{2}$ are computed empirically from a dataset. Note, $\lambda_{{m_{1} }} = N\left( {m_{1} } \right).$

This method of tsunami hazard assessments differs from other techniques because the kinematic parameters of the seismic source are incorporated in the tsunami variability. Rupture velocity can be included in different ways, for instance, expanding the interval of possible values with other probability distribution, accounting for less likely slow events, or simply to be set as infinite (static displacement), which retrieves common methods of seismic source sampling.

### Case study: Kuril-Kamchatka trench

**Figure 4:**
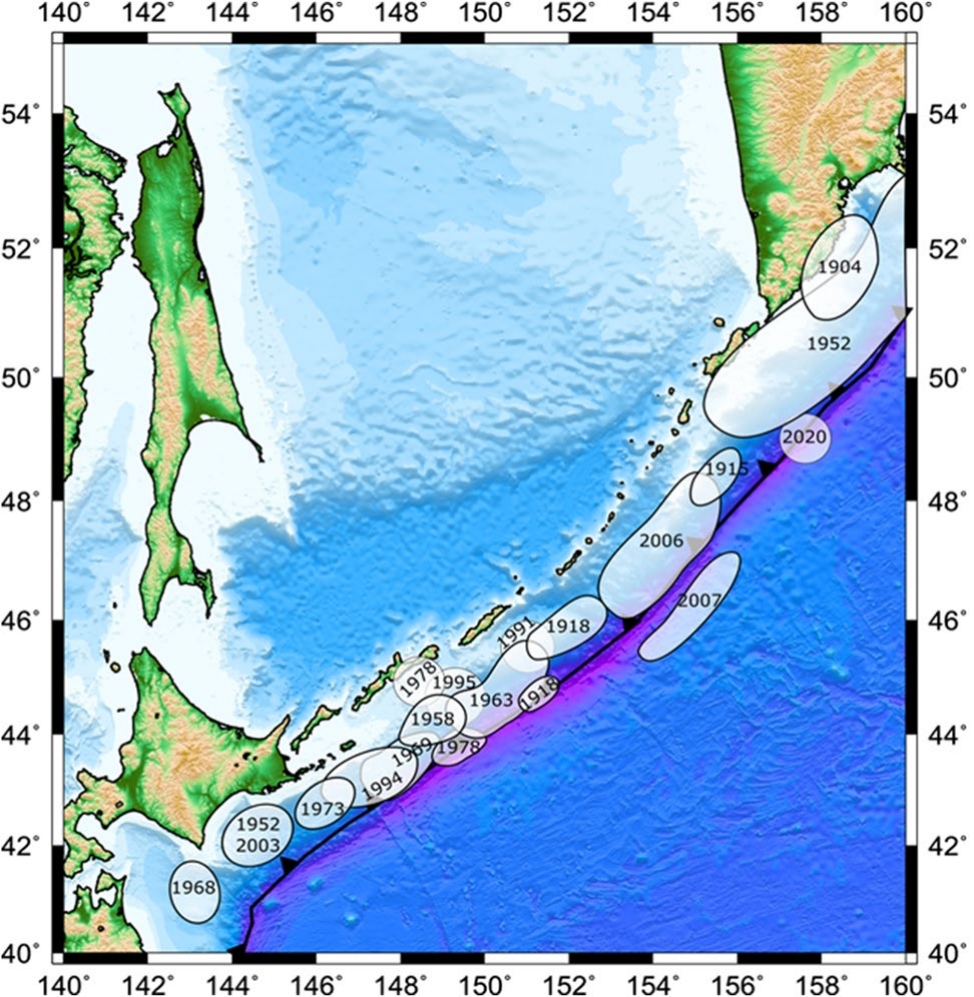
Significant earthquakes occurred in the Kuril-Kamchatka trench. The events of 1978, 1991 and 1995 do not have a finite fault estimation, however they are included in the seismic moment calculation. (Figure created with GMT 6.4.0, https://www.generic-mapping-tools.org/).

The Kuril—Kamchatka trench has experienced some of the most emblematic and large earthquakes recorded (Fig. 4).

Hirose et al.^41^ computed maximum earthquake along the Japan—Kuril—Kamchatka trench for different models, namely, G-R law, truncated G-R law, Utsu’s formula, Gamma distribution, Tapered G-R. Here, we take the truncated G-R law, because according to the authors, it presented better fits. Also, we restrict the following analysis to subduction earthquakes only. Crustal and outer-rise events are not considered since those events are less likely (but not impossible) to excite tsunami waves. Nonetheless, studied such as Mulia et al.^42^ provide models to include occurrence rates of active faults in the eastern margin of the Sea of Japan. Evidently, if these types of earthquakes are included, the final combined annual rates would change, but should not be comparative respect to subduction events, since the annual rate that produces significative tsunami amplitudes from active faults are two orders of magnitude smaller in Japan, according to Mulia et al.^42^.

We used the USGS seismic catalog completed with the compilation made by Gusev & Shumilina^43^. The parameters were estimated with a non-linear square method, weighted by the seismic moment.

**Figure 5:**
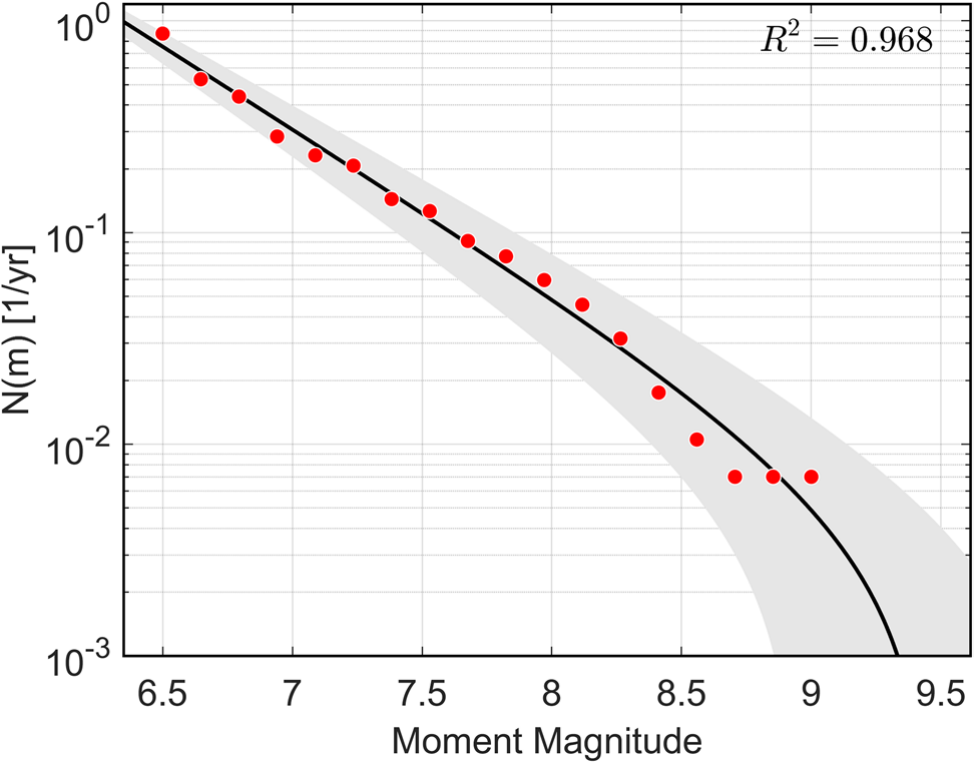
Truncated Gutenberg-Richter law fitted from data. Error band is also displayed in gray.

Then, with $m_{1} = 6.5,$ for a time window from 1737 to 2021 ($\Delta T = 285$), the obtained parameters were: $\lambda_{{m_{1} }} = 0.7554 \left( {0.6260, 0.8848} \right)$, $b = 0.7775 \left( {0.6881, 0.8670} \right)$ and $m_{2} = 9.464 \left( {8.953, 9.975} \right)$. Confidence intervals were computed at 95% of confidence (Fig. 5).

**Figure 6:**
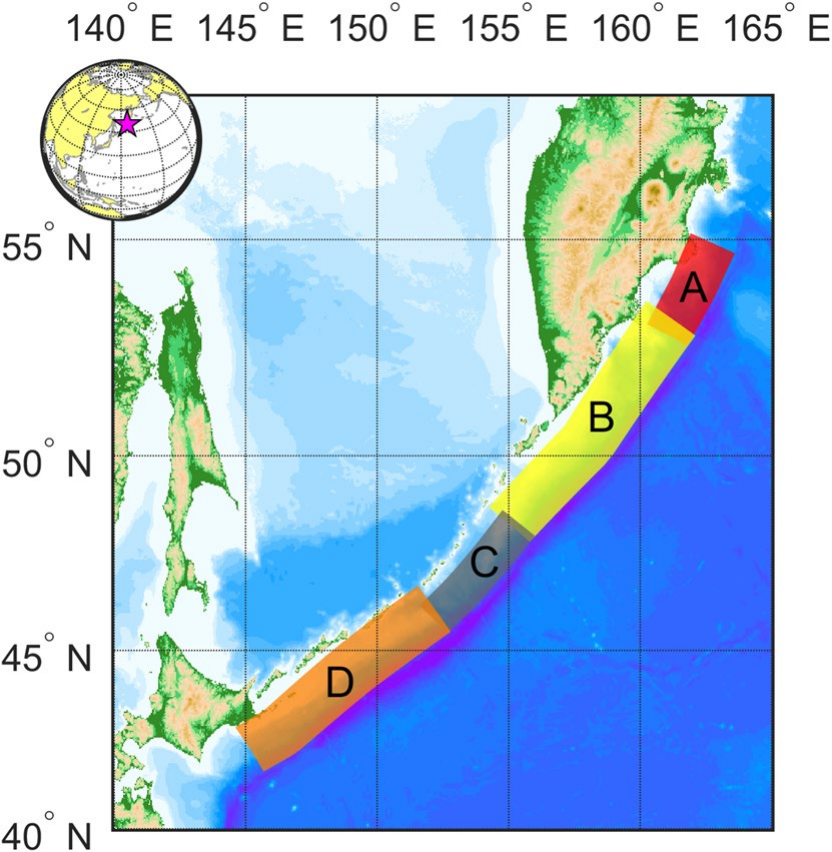
Segmentation of the Kuril-Kamchatka trench. Four segments are identified as potential areas where large earthquakes may occur. (Figure created with MATLAB 2022a, https://www.mathworks.com/).

We divide the Kuril-Kamchatka trench in 4 zones based on the available seismic moment in this seismogenic zone (Fig. 6) that represents four seismic gaps. There is a gap located between Simushir Island and Onekotan Island that has produced just one major earthquake since 1780**;** the Mw 8.3 earthquake on November 15, 2006, the event had an inverse mechanism^44^. There were aftershocks breaking one or more faults near the outer seaward uplift region of the Kuril-Kamchatka trench, adjacent to the location of the main shock. On January 13, 2007, another earthquake (Mw 8.1) occurred in this same region, parallel to the rupture of the 2006 earthquake with a normal mechanism. Because the event broke within the aftershock zone of the November 2006 earthquake, it is speculated that it is the result of changes in the regional stress field after the previous event despite occurring in a different fault^44^. On the other hand, it is observed that not all the area along the arc was broken meaning that there would be two sections that could generate ruptures in the near future.

Therefore, we identify the following seismic gaps. The first one is located between the Hokkaido and the Simushir Islands (zones C and D). The area available is located between the seventeenth century event and the Mw 8.3 event. The second one is located between the rupture area of the November 2006 earthquake (Mw 8.3) and the 1952 Mega-earthquake (Mw 9.0) which should be the largest earthquake in this area (zones A and B).

Additionally, it can be divided into two different situations, exempting the 1994 earthquake (Mw 8.3) with a depth of 14 km, because the fault rupture occurred in the upper part of the subduction interface (zone A).

The 1994 earthquake was an intraslab event and therefore did not reach the trench, so the tsunami threat remains high there in that area. There is a lack of information about the northern part of the Kuril Islands trench. Historically, the Kuril Islands have been the subject of territorial disputes between Japan and Russia, and even the border between the two countries is not fixed (https://www.mofa.go.jp/region/europe/russia/territory/overview.html). Therefore, Japan and Russia have not been able to conduct a joint survey of tsunami traces in the Kuril Islands. There is still a possibility that the D segment includes the southern part of the Kuril Islands (Kunashir, Iturup, Urup) and part of the subduction interface in the Hokkaido segment. For that reason, using scaling laws (as described earlier) a Mw 9.0 earthquake is plausible in the D segment.

Finally, combining occurrence rates (Table 1) and marginal probabilities from the tsunami numerical simulation, it is possible to construct hazard maps.

**Table 1:** Summary of earthquake rates of target magnitudes. Return periods are expressed in years and occurrence rate in events per year.

Zone	${M}_{w}$	$\lambda ({e}_{m})$	${T}_{R}({e}_{m})$
A	8.5	0.01738	58
B	9.0	0.00487	205
C	8.3	0.02649	38
D	9.0	0.00487	205

Strictly speaking, more magnitudes should be included in each zone. However, as more magnitude are included, the number of necessary scenarios increase exponentially. To simplify this problem, we take only the worst case by considering the maximum likely event. Note that Eq. (2) allows to include as many magnitudes as desired, by adding more terms in the inner sums.

For this example, the average rupture velocity is taken from the interval [2.0; 2.5] (km/s), and for the rake field, $\alpha_{0} = 90^\circ$ and $\alpha_{\sigma } = 30^\circ$.

To compute $P\left( {I\left( {x|e} \right) > I_{0} } \right)$, we generated 10.000 sources in each zone, according to the methodology inMethodology. Due to the numerical cost of the tsunami simulation, we select a subsample, keeping the statistical power.

The subsampling method is posed as follows: we take 3 statistics, in this case, the maximum slip $s_{m }$ and the slip standard deviation $\sigma_{s}$ accounts for spatial variability, and the centroid time $t_{m }$ accounts for temporal variability. Assuming that the set of 10.000 sources per zone represents a complete space, we define a confidence parameter $\in \left( {0,1} \right)$, and a $\in - M$ subsample, as a random subsample from the original sets of 10.000 sources, such as.

$$\rho \left( {F_{i}^{M} , F^{\infty } } \right) < \in$$,where

$$\rho \left( {x_{i} ,y} \right) = \sqrt {\mathop \sum \limits_{i = 1}^{Ns} \frac{{\left| {\left| {x_{i} - y} \right|} \right|^{2}_{{L^{2} }} }}{{\left| {\left| y \right|} \right|^{2}_{{L^{2} }} }}}$$

**Figure 7:**
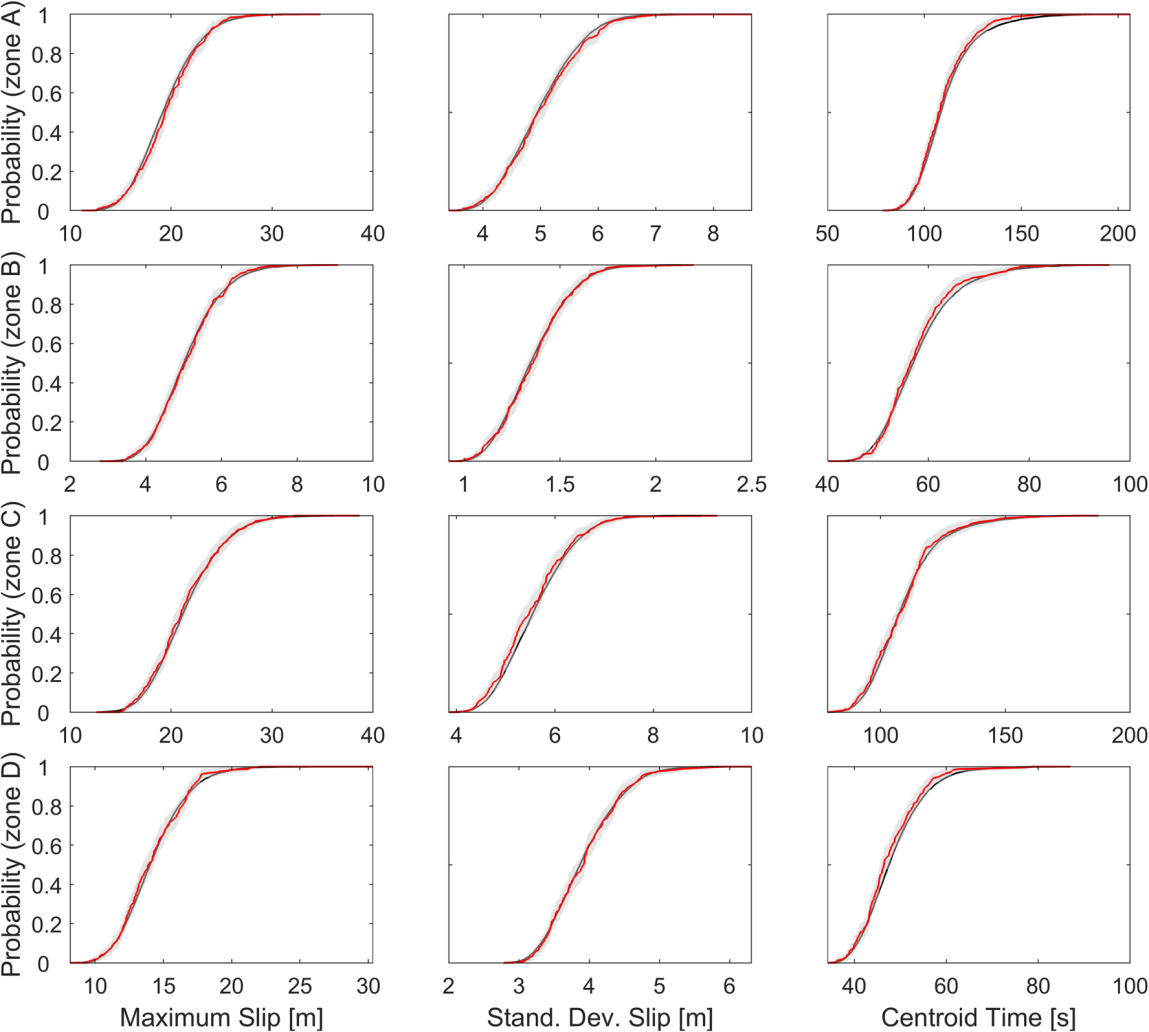
Statistic performance of the $\epsilon -M$ subsample. For $\epsilon =0.05$ and $M=250$, the probability distributions are close enough (within a $\epsilon$ tolerance) to their convergence limits.

**Figure 8:**
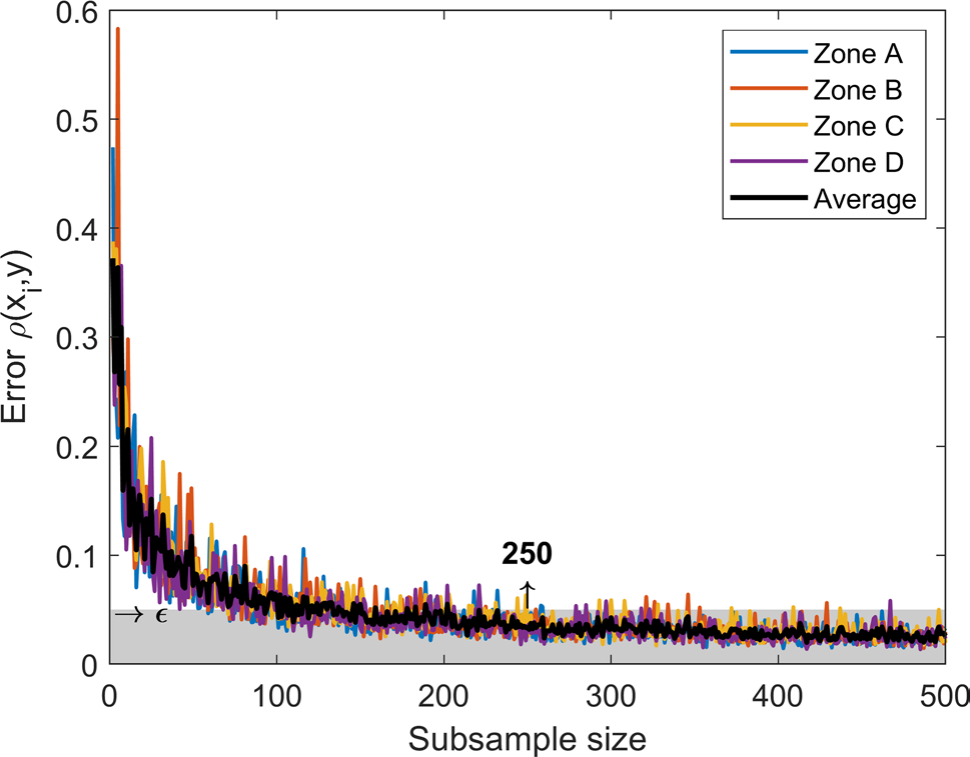
Convergence of the subsample at a given uncertainty level.

$N_{s}$ is the number of statistics (in this case, $N_{s} = 3$) , $M$ is the subsample size that ensures the convergence condition and $F^{k}_{i }$ is the empirical cumulative distribution function of the $i$-th statistic with $k$ terms. Figure 7 shows the case of a $\in - M$ subsample with $\in = 0.05$ and $M = 250$. This means that only 250 scenarios per zone are required to capture the same properties as the whole sets, trading off an uncertainty at most of $\in$ (Fig. 8).

The subsampling is tested by increasing the sample size up to 500. Each sample is randomly selected. Figure 8 shows the error metric $\rho \left( {x_{i} ,y} \right)$, exhibiting a decreasing behavior that tends to become slow after the size 250. This suggests that by increasing the subsample size, produces little effect on reducing the uncertainty at high computational costs.

Once the subsample is selected, tsunami simulation is performed for each scenario from the subsample. For each scenario, NEOWAVE requires two main inputs: the source and bathymetry. Bathymetry is nested until reaching a sufficiently small grid size to compute the inundation process. To illustrate this study case, we use the global GEBCO bathymetry charts of 15 arcsec of resolution in combination with the global SRTM topography of 1 arcsec. Unfortunately, no other specific local nautical charts were available around the Severo-Kurilsk town. The four digital elevation models are:

- level 1: [140.00, 165.00] x [40.00, 58.00] at 1 arcmin
- level 2: [155.50, 157.00] x [49.75, 51.25] at 15 arcsec
- level 3: [156.00, 156.50] x [50.30, 50.80] at 5 arcsec
- level 4: [156.10, 156.20] x [50.64, 50.74] at 1 arcsec

Therefore, all the components needed to evaluate the probabilities of exceedance are defined.

## Results

Exceedance probability can be evaluated directly from Eqs. (2) and (3), by picking an excess value and an exposure time.

**Figure 9:**
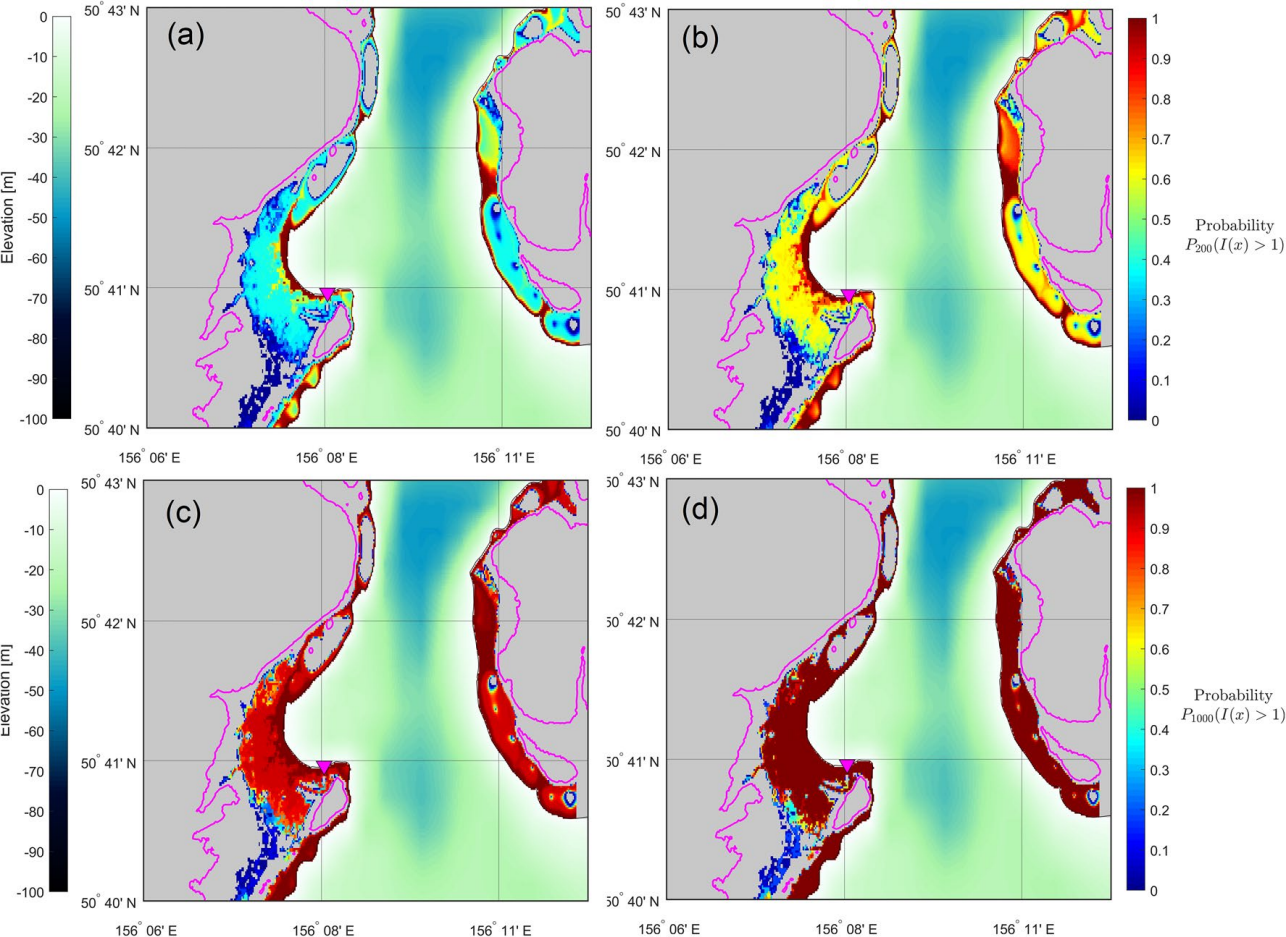
Hazard maps for flow depth exceedance of 1.0 m, for 100, 200, 500 and 1000 years of exposure time. Magenta curves show the 30 m contour level, for reference. (Figure created with MATLAB 2022a, https://www.mathworks.com/).

**Figure 10:**
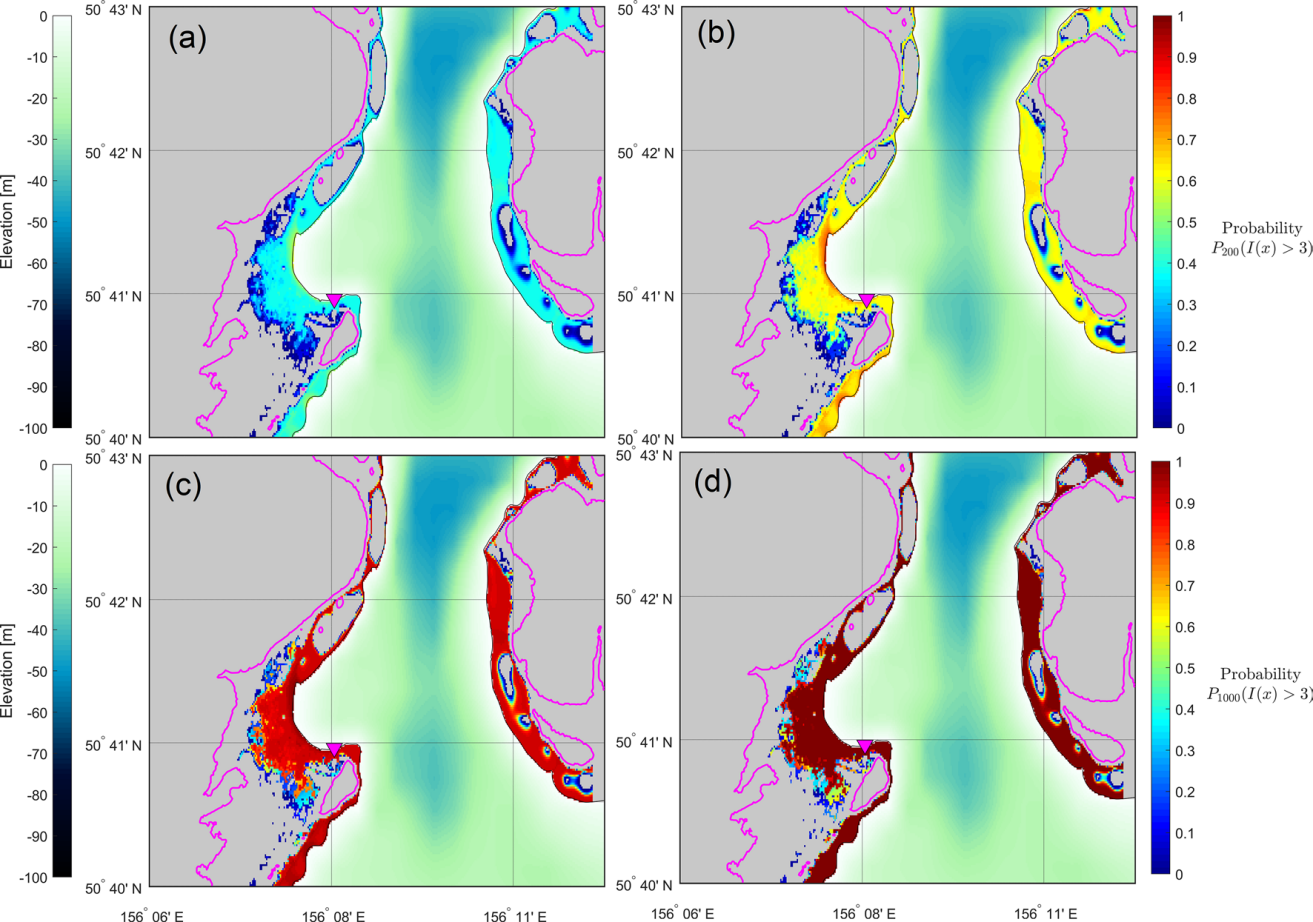
Hazard maps for flow depth exceedance of 3.0 m, for 100, 200, 500 and 1000 years of exposure time. Magenta curves show the 30 m contour level, for reference. (Figure created with MATLAB 2022a, https://www.mathworks.com/).

The study-case of Severo-Kurilsk shows significant hazard (Figs. 9, 10).

**Figure 11:**
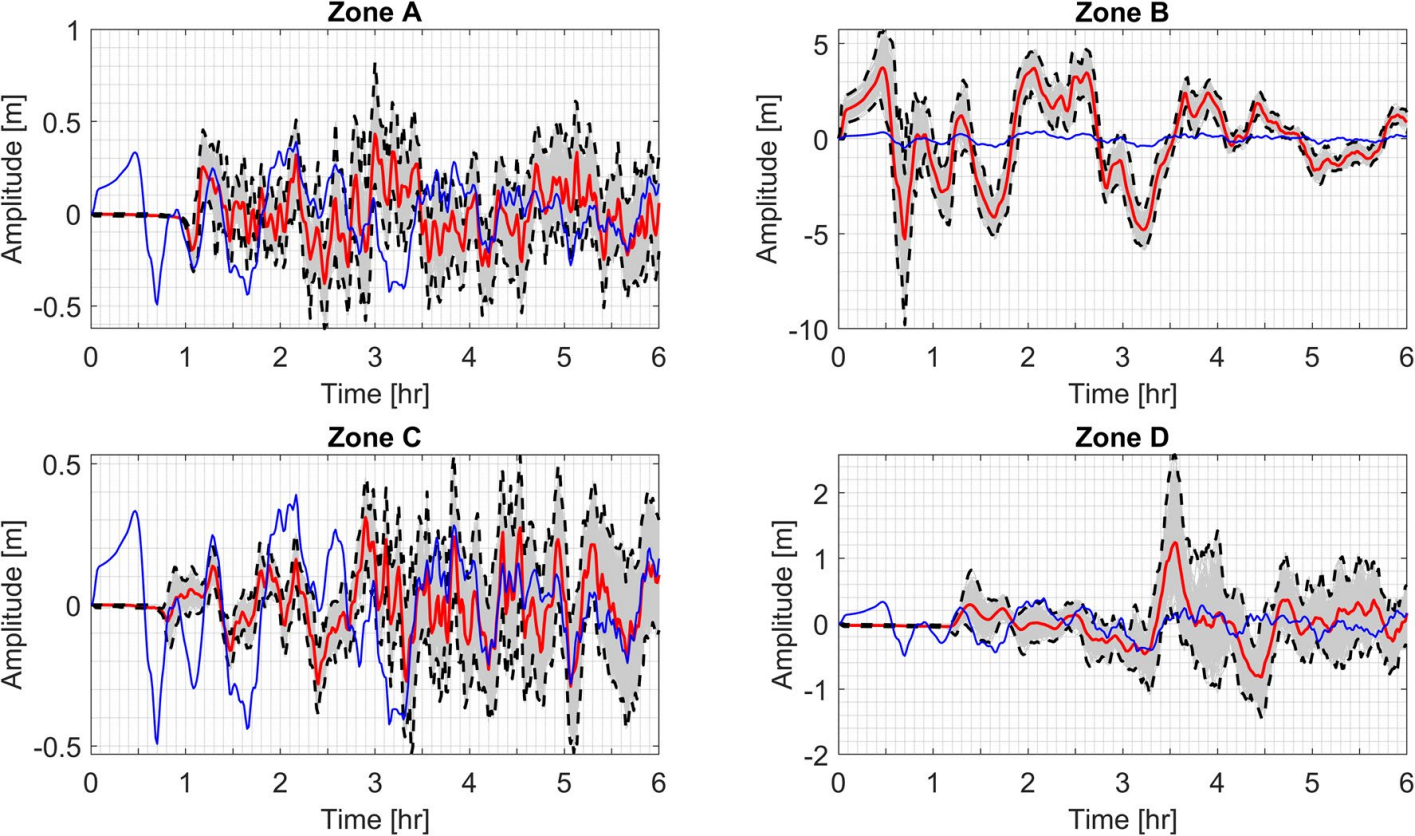
All time-series of the tide gauge at the port of Severo-Kurilsk by zone. Red line shows the average record and dashed lines are the upper and lower envelopes for each zone. The common blue record represents the global weighted (by recurrence rates) average tide gauge record Eq. (6).

Probability maps show the hazard at different locations at certain levels of exposure time. At each point, it is possible to analyze deeper. For instance, the tide gauge at the port of Severo-Kurilsk shows amplitude variability depending on the activated segment. This variability can be summarized as an upper and lower envelopes accounting for the corresponding uncertainty. Also, around 3–4 waves are expected within the first two hours (Fig. 11). The weighted average time series for the tide gauge is computed as

6 $$\frac{{\mathop \sum \nolimits_{e} \lambda \left( e \right)\frac{1}{M}\mathop \sum \nolimits_{i = 1}^{M} \eta_{i}^{e} \left( t \right)}}{{\mathop \sum \nolimits_{e} \lambda \left( e \right)}}, e \in \left\{ {A,B,C,D} \right\}$$

where $\eta_{i}^{e} \left( t \right)$ is the time series of the tide gauge of the scenario $i$ in the zone $e$.

**Figure 12:**
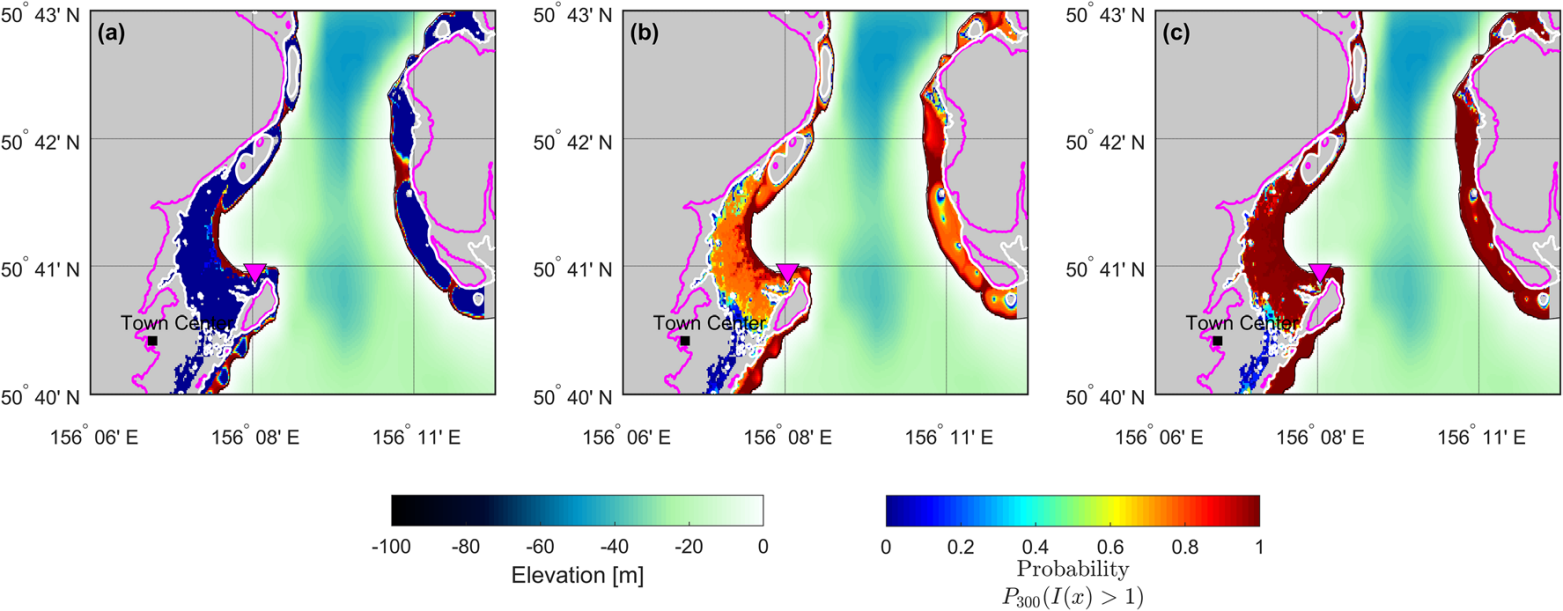
Hazard map for flow depth exceeding 1.0 m in the next 300 years (middle). Lower and upper bounds for probabilities are shown (left and right, respectively). Magenta curves show the 30 m contour level, and white curves show the 10 m contour level. (Figure created with MATLAB 2022a, https://www.mathworks.com/).

This modeling suggests that the Severo-Kurilsk town, after the previous tsunamis, still exhibits significant hazard based on the probability results, especially in coastal zones, below 10 m. In fact, average inundation limit (run-up height), is close to contour level of 10 m (Fig. 12).

Since probability is associated with uncertainties due to multiple sources, such as the parameter estimation of the G-R law and the subsampling process, which includes the seismic source uncertainties, lower and upper bounds of the imprecise probabilities are computed, as shown in Fig. 12. This helps to visualize the zones where uncertainties are larger. For instance, variability is smaller close to the coastline close to the limit of the maximum inundation level.

**Figure 13:**
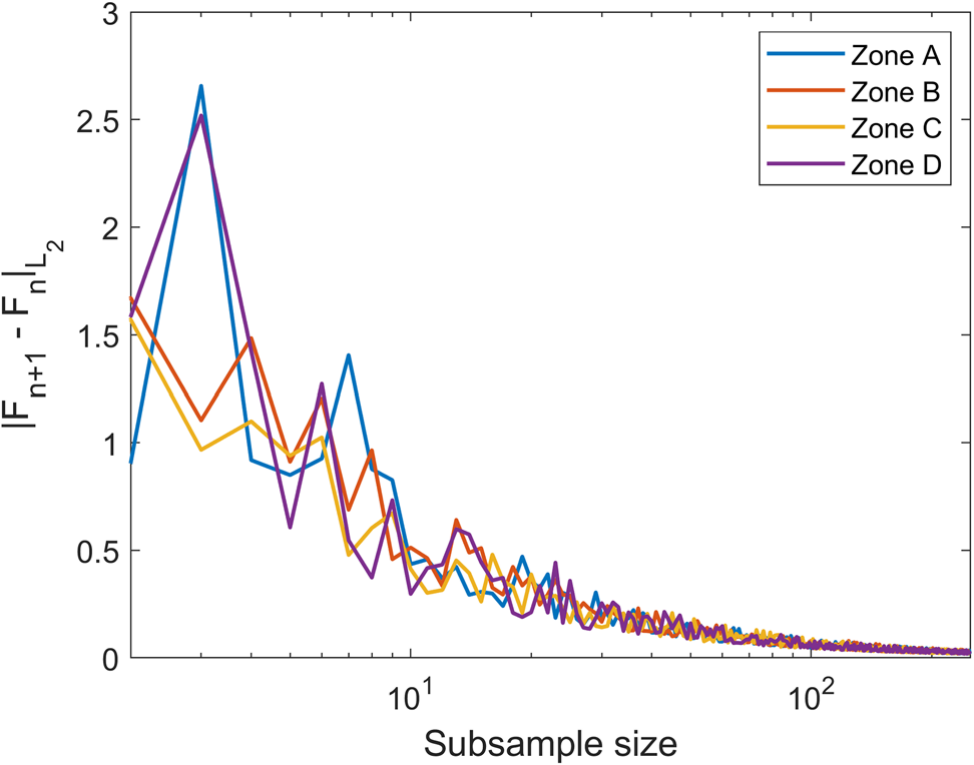
Relative difference sequence of the ecdf of the tide gauge at the port of Severo-Kurilsk. Each zone converges to a final ecdf fast enough associated with the subsample size.

To verify the performance of the sampling technique, we define $F_{n} \left( x \right) = P\left( {\mathop {\max }\limits_{t > 0} \eta_{port} \left( t \right) < x} \right)$ as the empirical cumulative distribution function (ecdf) of the maximum amplitude of the coastal tide gauge located in the port of Severo-Kurilsk, for a sample of $n$ scenarios. We compute the relative difference by adding an extra scenario to the subsample per iteration. Figure 13 shows a quick convergence (at least in the Cauchy sense), to a final ecdf.

Other techniques for avoiding a large set of tsunami simulations are described by Sepulveda et al.^45^ and Davies et al.^46^. Stochastic Order Reduction methods or efficient Monte Carlo approaches are also valid alternatives to treat a large dataset of scenarios.

## Conclusions

A new stochastic seismic source model is presented. This approach is employed to evaluate tsunami hazard. However, it can also be used in other fields, such as seismic hazard and risk or, to create synthetic seismograms.

In the case of tsunami modeling, the proposed methodology is simple and direct to apply, as long as data is available, and can be applied everywhere that is exposed to tsunamigenic earthquakes. The inclusion of more realistic seismic sources allows to reduce systematic uncertainties. For instance, kinematic parameters, hypocenter location and rake variability increase the sources of uncertainties. De Risi and Goda^33^ suggested 200 sources to cover spatial variability (for a Mw 9.0 earthquake), but in this study, 250 were needed to fulfill the required level of uncertainty tolerance, which can be tuned to diminish the number of computed scenarios. Future studies might focus on deeper comparison with previous methods in order to quantify the differences in the final hazard product.

Inundation charts can be calculated in probabilistic terms, which is more suitable for coastal planning and insurance premium deductions. Nevertheless, many sources of uncertainties are still difficult to assess. For example, the segmentation of the trench is hard to define, especially in areas poorly constrained with seismic history. For future works, we suggest investigating the influence of the segmentation, where different locations and sizes of the sources should be tested. Other source of uncertainties comes from the geometry fault parameters. In this case, we used the slab 2.0 model which offers an uncertainty band of the depth but still is difficult to characterize other parameters such as dip and strike, and they are usually fixed. We suggest focusing on these aspects in future investigations. Also, more statistics should be tested in order to verify the stability of the convergence of the subsampling process. However, this might require more computing resources.

On the other hand, with the observation capabilities around seismic networks, extreme events are being observed. Using this method, it would be possible to study very slow earthquakes that trigger tsunamis. Recently, Jia et al.^47^ have shown that the 2021 South Sandwich Island Mw 8.2 Earthquake had a very slow rupture velocity. This means that propagation and inundation can reach higher values than those earthquakes with regular rupture velocity^30^ and therefore is important to include to this parameter as more observations are collected.

As more physical parameters are included, new sources of uncertainties appear, and the methodology relies on a huge set of numerical scenarios being limited by computational resources. Specific comparison with static scenarios is also recommended in future studies.

## Data Availability

The datasets used and/or analyzed during the current study are available from the corresponding author on reasonable request.
